# An Unusual Case of Blackout in a COVID-19 Patient: COVID-19 Brain Fog

**DOI:** 10.7759/cureus.36273

**Published:** 2023-03-17

**Authors:** Lareb Hassan, Zainab Ahsan, Hunniya Bint E Riaz

**Affiliations:** 1 Internal Medicine, Quaid-e-Azam Medical College, Bahawalpur, PAK; 2 Internal Medicine, Combined Military Hospitals (CMH) Lahore Medical College and Institute of Dentistry, Lahore, PAK

**Keywords:** guillain-barré syndrome (gbs), covid 19, coronavirus disease 2019 (covid-19), sars-cov-2, cytokine storm, neurological implications, brain fog, long covid

## Abstract

This case report highlights a unique case of brain fog in a COVID-19 patient suggesting COVID-19's neurotropic nature. COVID-19 is associated with a long‐COVID syndrome that presents with cognitive decline and fatigue. Recent studies show the emergence of a novel syndrome known as post-acute COVID syndrome or long COVID, which constitutes a variety of symptoms that continue for four weeks following the onset of a COVID-19 diagnosis. Numerous post-COVID patients experience both short and long-lasting symptoms affecting several organs, including the brain, which includes being unconscious, bradyphrenia, or amnesia. This long COVID status comprises of "brain fog", which, coupled with neuro-cognitive effects, has a significant role in prolonging the recovery phase. The pathogenesis of brain fog is currently unknown. One of the leading causes might be the involvement of neuroinflammation due to mast cells stimulated by pathogenic and stress stimuli. This in turn, triggers the release of mediators that activate microglia, causing inflammation in the hypothalamus. Its ability to invade the nervous system through trans-neural or hematogenous mechanisms is possibly the chief cause behind the presenting symptoms. This case report highlights a unique case of brain fog in a COVID-19 patient suggesting COVID-19's neurotropic nature and how it may lead to neurologic complications such as meningitis, encephalitis, and Guillain-Barré syndrome.

## Introduction

The severe acute respiratory syndrome coronavirus 2 (SARS-CoV-2) or coronavirus disease 2019 (COVID-19) pandemic has caused unprecedented morbidity and mortality worldwide all over the world after the World Health Organization (WHO) declared it a global pandemic in 2020 [1]. Co-morbidities in COVID-19 patients, such as diabetes, hypertension, bacterial infection, and immunosuppression, have been associated with increased hospitalization and intensive care unit admissions relative to non-hospitalized patients [2]. COVID-19 is a positive-sense, enveloped, single-stranded, ribonucleic acid (RNA) virus belonging to the coronaviridae family. It manifests as a cough, fever, anosmia, myalgia, dyspnea, fatigue and pharyngitis, headache, and diarrhea as lesser uncommon symptoms [3]. This viral infection can induce cellular damage through a strong, robust innate immune response via inflammatory cytokine storm and establishing a pro-coagulant state that may contribute to these sequelae and residual symptoms later [4]. Apart from causing respiratory and systemic symptoms, a broad spectrum of neurologic complications that includes meningitis, encephalitis, myelitis, acute disseminated encephalomyelitis, Guillain-Barré syndrome, metabolic and acute hemorrhagic necrotizing encephalopathy, cerebrovascular diseases polyneuritis cranialis, dysautonomia, and myopathies, have been documented reported in COVID-19 patients [5]. This essentially demonstrates that the SARS-CoV-2 virus can either invade and dominate the nervous system either through trans-neural or hematogenous mechanisms [6]. Abnormal coagulation profiles such as increased D-dimer, fibrin/fibrinogen degradation products, and mild thrombocytopenia manifesting associated with a prothrombotic state, leading to stroke and other cerebrovascular accidents, have been reported in COVID-19 patients as well [7,8]. Recent studies show literature suggests the emergence of a novel syndrome known as post-acute COVID syndrome or long COVID, a term that comprises a diverse variety of sets of multiorgan symptoms that continue for four weeks after the diagnosis of COVID-19 [9]. after a minimum of four weeks from the onset of a diagnosed COVID-19 infect clinical signs of long COVID syndrome include fatigue, dyspnea, myalgia, diffuse pain, headaches, anxiety/depression, and cognitive impairments (brain fog) [10].

Below is a case report of a patient suffering from brain fog after COVID-19 infection.

## Case presentation

A 24-year-old COVID-19-recovered female presented in the outpatient department (OPD) with a complaint of loss of consciousness on the 18th day, post-quarantine at home. The patient reported symptoms of conjunctivitis and a 104 F fever. These were accompanied by pleuritic pain, headache, sore throat, fatigue, myalgia, phlegm, and runny nose. The patient self-medicated with Tylenol twice a day and steroid eye drops. COVID-19 was diagnosed through a nasopharyngeal swab previously. After receiving positive polymerase chain reaction (PCR) results, the patient followed medicine prescription via telemedicine that included a regimen of Tylenol, azithromycin, ebastine, montelukast, Provas-N, multi-vitamins, Cal-C tablets, and a cough expectorant. Her oxygen saturation never dropped below 92% at a home pulse oximeter. On the 18th day of her quarantine, she felt generalized weakness succeeded with a fall at ground level during ambulation. This was preceded by tremors, blurry vision, and a generalized ascending pattern. She was reportedly woken up by a family member an hour later. She had no previous history of any neurological symptoms or seizures. However, she has had a few hypoglycemic fainting spells in the past due to inadequate nutrition. This episode, however, was just after eating a meal. She reported at the OPD that the fever had already resolved, oxygen saturation was normal, and there were no neurologic deficits. The general physical examination was unremarkable as well. She showed signs of recovery despite the neurologic incident. The patient's complete blood count report was within the normal range (Table 1). The COVID-19 antibody test was reactive. She had not received any vaccination for COVID-19 prior to testing positive.

**Table 1:** Laboratory findings of the COVID-19 patient COVID-19 - coronavirus disease-19

Blood component	Abbreviation	Patient’s values	Reference range
White blood cells	WBC	6.87	4500-11,000/mm^3^
Red blood cells	RBC	4.62	Male: 4.3-5.9 million/mm^3^ Female: 3.5-5.5 million/mm^3^
Hemoglobin	HGB	12.7	Male: 13.5-17.5 g/dL Female: 12.0-16.0 g/dL
Hematocrit	HT	39.4	Male: 41%-53% Female: 36%-46%
Mean corpuscular volume	MCV	85	80-100 µm^3^
Mean corpuscular hemoglobin	MCH	27.5	25.4-34.6 pg/cell
Mean corpuscular hemoglobin concentration	MCHC	32.2	31%-36% Hb/cell
Platelets	Platelets	305000	150,000-400,000/mm^3^
C- Reactive protein	CRP	<0.3	0.3 to 10 mg/L

## Discussion

This case report illustrates a case of brain fog in a COVID-19 patient, likely due to a neurological pathology. The most common persistent neurologic symptoms observed in non-hospitalized COVID-19 'long haulers' are found to be brain fog (81%) and paresthesia numbness/tingling (60%) [11]. The pathogenesis of brain fog in this illness is currently unknown. Still, present studies suggest the involvement of it might involve neuroinflammation via mast cells triggered by pathogenic and or stress stimuli resulting in the release of mediators that activate microglia and lead to inflammation of thein the hypothalamus [12]. As per current literature, it has been predicted hypothesized that COVID-19 infection affects the autonomic nervous system [13]. The association between the two is multifaceted: the well-documented cytokine response storm of COVID-19 occurs through the activation of the sympathetic system because sympathetic activation results in induced pro-inflammatory cytokine release, which produces a 'cytokine storm' [14,15]. Orthostatic hypotension is also observed in some patients presenting with similar neurologic symptoms. Another follow-up symptom presented due to disturbance in the autonomic nervous system in long COVID is orthostatic intolerance; however, besides encompassing many other symptoms, more research is required to co-relate the episodes of brain fog to COVID patients experiencing orthostatic hypotension in COVID patients [16]. With emerging cases, a variety of diverse sets of neurological symptoms and complications have been documented and presented. Neurological complications that have been reported amongst patients range from mild headache, hypogeusia, hyposmia, and impaired unconsciousness to severe or critical disease states like encephalopathy, stroke, Guillain-Barre Syndromé (GBS), central nervous system (CNS) demyelination, GBS infarcts, microhemorrhages, and nerve root enhancement [17,18]. An evolving neurological disease of GBS is also known to be reported in COVID patients. A case report highlighted such a case of progressive paraparesis, areflexia, and paresthesia [19]. Sensory loss with tingling of all extremities found in a patient. The patient under discussion was also in the present case report of brain fog reported with ascending numbness in a caudocranial fashion. The current patient reports symptoms of tremors at the beginning of her brain fog episode. So far, only smaller cohort studies or single cases have reported cerebrovascular events, seizures, meningoencephalitis, and immune-mediated neurological diseases in patients, which are not suitable for quantitative analysis. Amongst these symptoms, COVID's effect on the neurological system is evident [20]. The patient also reported having dinner before her fainting episode, ruling out hypoglycemia as a potential cause. While current evidence surrounding COVID-19's effect on neurological symptoms is progressive, prelusive data has suggested a significant correlation.

Limitations

High-resolution computed tomography (HRCT) and chest X-ray were not done. Cerebrospinal fluid (CSF) examination, magnetic resonance imaging (MRI), and Doppler ultrasound should be conducted in order to elucidate the correlation of the neurological episode of brain fog with COVID-19. The duration between the fainting episode and the patient reporting to the doctor was 18 days. Lab tests done immediately after the incident and progressive monitoring done by the hospital staff would have indicated more clear explanations.

## Conclusions

This case report highlights an unusual case of brain fog in a COVID-19 patient suggesting COVID-19's neurotropic nature and how it might have neurological implications because of it. This case report shows the importance of being aware of atypical neurological findings for the early diagnosis and treatment of COVID-19 patients. We report that despite experiencing neurological symptoms, the patient showed no clinical findings suggestive of neurological damage. Additional, comprehensive longitudinal case studies are needed to help create concrete support for the impact of COVID-19 on the nervous system.
